# STHMA: Decoupling Spatio-Temporal Dynamics in EEG via Hybrid State Space Modeling

**DOI:** 10.3390/brainsci16030267

**Published:** 2026-02-27

**Authors:** Shuo Yang, Lintong Zhang, Youyi Cheng, Yingying Zheng, Shuai Zheng, Jiahui Guo, Lirong Zheng

**Affiliations:** 1 School of Biomedical Engineering, Southern Medical University, Guangzhou 510515, China; y17734063493@i.smu.edu.cn (S.Y.); zlt@i.smu.edu.cn (L.Z.); 3208010137@i.smu.edu.cn (Y.C.); z32411@i.smu.edu.cn (Y.Z.); 2 Guangdong Institute of Intelligence Science and Technology, Guangdong-Macao In-Depth Cooperation Zone in Hengqin, Zhuhai 519031, China; 3 College of Future Information Technology, Fudan University, Shanghai 200433, China; zhengshuai@fudan.edu.cn; 4 Department of Psychology, School of Social Development and Public Policy, Fudan University, Shanghai 200433, China

**Keywords:** Human–Computer Interaction, signal processing, affective computing, deep learning

## Abstract

**Highlights:**

**What are the main findings?**

- The proposed STHMA framework achieves state-of-the-art emotion recognition performance on the FACED and SEED-V datasets, outperforming Transformer-based baselines by effectively combining linear-complexity State Space Models with global attention mechanisms.
- Ablation studies demonstrate that the “Decoupled Spatial–Temporal Scanning” strategy—which alternates between modeling instantaneous brain connectivity and continuous temporal dynamics—is the most critical component for reconstructing the complex spatio-temporal manifold of EEG data.

**What are the implications of the main findings?**

- The results validate that modeling physiological signals as continuous dynamical systems via State Space Models offers better representational fidelity than the discrete tokenization used in Transformers, resolving theoretical mismatches in biological signal processing.
- The architecture’s linear computational complexity overcomes the scalability bottlenecks of traditional attention mechanisms, enabling the development of real-time Brain–Computer Interfaces capable of processing long-duration high-resolution neural recordings.

**Abstract:**

**Background/Objectives:** Decoding affective states from Electroencephalography (EEG) signals is fundamental to non-invasive Brain–Computer Interfaces. Despite recent advances, accurate recognition is impeded by the inherently non-stationary nature of physiological signals and the entanglement of spatio-temporal dynamics within high-dimensional recordings. While Transformers excel at global modeling, they often neglect the continuous dynamical properties of neural signals and suffer from quadratic complexity. **Methods:** In this paper, we propose the Spatio-Temporal Hybrid Mamba-Attention (STHMA), a framework designed to explicitly disentangle and model EEG dynamics via linear-complexity State Space Models. First, to incorporate domain knowledge, we introduce a Dual-Domain Physics-Aware Embedding module. This module fuses learnable temporal convolutions with explicit frequency-domain spectral features, ensuring fidelity to neurophysiological principles. Second, we propose a novel Decoupled Spatial–Temporal Scanning strategy. By dynamically reconfiguring the serialization of the data tensor, our model strictly separates the learning of instantaneous functional connectivity from the tracking of emotional state evolution, thereby preventing the structural collapse common in 1D sequence models. **Results:** Extensive experiments on the FACED and SEED-V datasets demonstrate that the STHMA achieves state-of-the-art performance, significantly exceeding the random chance baselines (11.11% for 9-class FACED and 20.00% for 5-class SEED-V). **Conclusions:** The results validate that combining Physics-Aware Embeddings with decoupled state-space modeling offers a scalable and effective paradigm for EEG emotion recognition.

## 1. Introduction

Emotion recognition constitutes a foundational pillar in Human–Computer Interaction (HCI) and neuro-healthcare, enabling systems to perceive and respond to human affective states [1]. Among various modalities, Electroencephalography (EEG) provides a direct, non-invasive window into the brain’s neural dynamics [2]. Unlike external cues such as facial expressions, which are susceptible to suppression, EEG signals offer a truthful representation of internal emotional processing [3]. However, robust EEG decoding remains a formidable challenge, primarily due to the signal’s inherent non-stationarity, low signal-to-noise ratio, and significant inter-subject variability that impedes generalization [4].

The fundamental difficulty in modeling EEG lies in the complex entanglement of its spatio-temporal manifold. The data exhibit two distinct forms of dependency: *Spatial Connectivity*, representing the functional synchronization among brain regions (electrodes) at a given instant; and *Temporal Dynamics*, representing the continuous evolution of neural states over time [5]. Traditional Deep Learning paradigms often struggle to unify these perspectives. Convolutional Neural Networks (CNNs) [6] excel at extracting local spatial patterns but lack the receptive field to model long-range temporal transitions. Conversely, Recurrent Neural Networks (RNNs) [7] suffer from gradient vanishing and prohibitively slow sequential processing, limiting their ability to capture fine-grained physiological fluctuations.

Recently, Transformers [8] have become the dominant paradigm in biological signal processing, leveraging Self-Attention to model global dependencies. Despite their success, applying Transformers to continuous physiological signals introduces a theoretical mismatch. The standard “Patchify” operation discretizes the continuous EEG waveform into independent tokens [9], potentially disrupting the underlying continuous dynamical system essential for describing neural activity [10]. Furthermore, the quadratic complexity ($O(N^{2})$) of attention maps restricts the modeling of high-resolution [11], long-duration temporal contexts necessary for analyzing complex emotional states [12].

To bridge this gap, Structured State Space Models (SSMs) [13,14] have emerged as a paradigm shift. Mathematically rooted in the discretization of continuous-time Ordinary Differential Equations (ODEs), SSMs naturally align with the continuous nature of biological signals while achieving linear computational complexity (O(N)) [15]. However, naively applying 1D SSMs to multi-channel EEG data is non-trivial [16]. Flattening the 2D EEG tensor (Channel × Time) into a 1D sequence inevitably introduces a *serialization ambiguity*—it disrupts either the spatial topology or the temporal continuity, depending on the scanning order, leading to suboptimal representation learning [17].

In this paper, we propose the Spatio-Temporal Hybrid Mamba-Attention (STHMA), a novel architecture designed to explicitly disentangle and model the spatiotemporal complexities of EEG signals. Our approach moves beyond simple application of SSMs by introducing three physically grounded architectural innovations:

First, we address the limitations of raw time-domain inputs by introducing a Dual-Domain Physics-Aware Embedding. We posit that emotional markers are distributed across both transient temporal waveforms and steady-state spectral power bands. Our embedding fuses learnable temporal convolutions with explicit Log-Magnitude FFT features, integrating neurophysiological domain knowledge directly into the deep learning pipeline.

Second, to overcome the topological loss in 1D serialization, we propose a Decoupled Spatial–Temporal Scanning strategy. Instead of a fixed flattening, our model dynamically reconfigures the data tensor between layers. It strictly separates the learning of instantaneous brain connectivity from the tracking of independent channel dynamics. By alternating between these orthogonal perspectives, the STHMA effectively reconstructs the spatio-temporal manifold without the quadratic cost of full attention.

Third, we recognize that while recurrent SSMs excel at tracking dynamics, they may struggle with global context comparison. We thus employ a hybrid architecture that culminates in a global Multi-Head Attention layer. This design combines the efficient, continuous modeling of Mamba with the global reasoning capability of Attention.

We evaluate the STHMA on the FACED [18] and SEED-V [19] datasets. The results demonstrate that our model achieves state-of-the-art performance, validating the efficacy of decoupling spatio-temporal dynamics via structured state space modeling.

In summary, the main contributions of this work are as follows:

- We propose the STHMA, a physics-informed architecture that adapts the continuous modeling capabilities of Mamba2 for EEG analysis, offering a theoretically superior alternative to discrete Token-based Transformers.
- We design a Dual-Domain Physics-Aware Embedding that explicitly fuses spectral features with temporal convolutions, mitigating the non-stationarity of EEG signals by anchoring representations in the frequency domain.
- We introduce a Decoupled Spatial–Temporal Scanning strategy that solves the “serialization ambiguity” of 1D SSMs, enabling precise modeling of both functional brain connectivity and dynamic emotional evolution.

## 2. Related Work

The trajectory of EEG decoding algorithms has evolved from manual feature engineering to end-to-end representation learning [20], driven by the need to capture the complex spatio-temporal dynamics of neural activities. We categorize the existing literature into three paradigms: Convolutional and Recurrent baselines, Transformer-based Global Modeling, and the emerging Structured State Space Models.

### 2.1. Local Feature Extraction

Early deep learning approaches primarily utilized Convolutional Neural Networks (CNNs) [21] to extract topological features from electrode grids [22]. Seminal works like EEGNet [23] introduced depthwise-separable convolutions to learn spatial filters efficiently, establishing a robust baseline for BCI tasks. Similarly, SPaRCNet [24] demonstrated that dense convolutional structures could capture fine-grained pathological patterns. To integrate temporal modeling, hybrid architectures cascading CNNs with Recurrent Neural Networks (RNNs) [25], such as LSTMs or GRUs, became prevalent [26]. For instance, FFCL [27] employs hierarchical structures to track time-varying characteristics.

However, these methods face inherent structural limitations. CNNs possess a strong *locality bias*; even with dilated kernels, their effective receptive field is insufficient to capture global brain connectivity [28]. Conversely, while RNNs theoretically model infinite history, they suffer from the “horizon problem”—gradient decay prevents the learning of emotional states persisting over long durations. Moreover, their sequential nature precludes parallel training, creating a computational bottleneck for high-resolution physiological data [29].

### 2.2. Global Modeling via Discrete Tokenization

To overcome the locality constraints of CNNs, the field shifted towards Transformer-based architectures, leveraging Self-Attention [30] to model global dependencies regardless of distance [31]. The EEG Conformer [32], ST-Transformer [33] and CNN-Transformer [34] exemplify this trend, combining local convolution with global attention to achieve state-of-the-art results. Other works, such as ContraWR [35], utilize Transformers for self-supervised contrastive learning to extract robust representations from unlabeled data.

Despite their dominance, Transformers introduce a theoretical mismatch when applied to biological signals. EEG signals are generated by continuous dynamical systems, whereas Transformers operate on discrete tokens. The standard “Patchify” operation [36] inevitably disrupts the waveform’s temporal smoothness and phase integrity. Furthermore, the quadratic complexity ($O(N^{2})$) [37] of the attention mechanism limits scalability. As sequence length [38] increases to capture extended emotional contexts, the memory footprint becomes prohibitive, hindering deployment in real-time BCI systems [39].

### 2.3. Continuous Dynamics and State Space Models

Recent advances in Structured State Space Models (SSMs), originating from the HiPPO theory [40] and formalized in S4 [41], offer a rigorous mathematical framework for modeling long-range dependencies in continuous time. By discretizing a continuous latent state evolution, SSMs combine the parallel training efficiency of CNNs with the inference speed of RNNs. The Mamba architecture [13,14] further introduces a data-dependent selection mechanism, enabling the model to selectively propagate relevant information over indefinitely long sequences with linear complexity (O(N)).

While Mamba2 [14] has revolutionized 1D text and genomic analysis, its application to multidimensional physiological signals remains underexplored. A fundamental challenge lies in the *serialization ambiguity*: EEG data possess a 2D topology (Space × Time). Naively flattening this tensor into a 1D sequence for Mamba2 [14] processing forces an arbitrary choice—either prioritizing spatial proximity at the cost of temporal continuity [42], or vice versa [17].

This work specifically addresses this gap. Unlike existing hybrids that merely stack modules, our STHMA proposes a *Decoupled Spatial–Temporal Scanning* strategy. We explicitly alternate between spatial and temporal views, allowing the 1D selective scan to reconstruct the 2D spatio-temporal manifold without the inductive bias trade-offs inherent in previous approaches.

## 3. Methods

**Figure 1:**
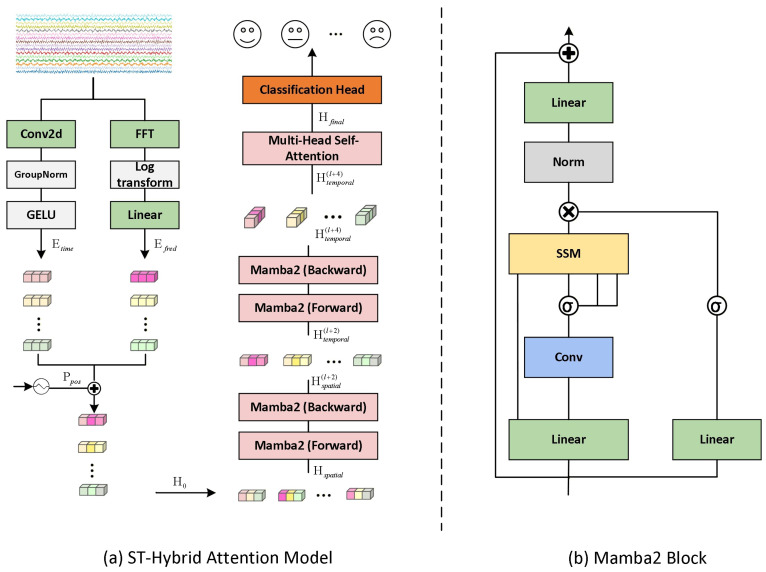
The pipeline of the STHMA for emotion recognition.

In this section, we present the proposed STHMA framework for EEG-based emotion recognition. The overall architecture is illustrated in Figure 1.

### 3.1. Dual-Domain Physics-Aware Embedding

Standard linear projections in Transformers often neglect the distinct spectral characteristics of physiological signals. To address this, we propose a *Dual-Domain Physics-Aware Embedding* that explicitly integrates temporal waveforms with spectral power distributions. Let $X\in R^{C\times T}$ denote a raw EEG trial, where *C* represents the number of electrodes (channels) and *T* is the number of discrete time points. The objective is to learn a mapping function $f_{\theta }:X\to \hat{y}\in Y$ that predicts the emotional state $\hat{y}$ from a set of classes Y. Due to the non-stationary nature of EEG, we first segment X into *N* non-overlapping patches, reshaping the input to a tensor $X_{in}\in R^{C\times N\times P}$, where *P* is the patch size.

#### 3.1.1. Temporal and Spectral Streams

We hypothesize that emotional markers are distributed across both transient temporal fluctuations and steady-state frequency bands.

- **Temporal Stream:** To capture local waveform semantics, we apply a depthwise-separable convolution $\phi_{time}$ along the time axis. Crucially, to normalize the variance of the raw temporal signals, we apply Group Normalization (GN) before activation. This compresses high-frequency noise into a latent semantic space:
  (1) $$E_{time}=\mathrm{GELU}\left(\mathrm{GN}\left(\phi_{time}\left(X_{in}\right)\right)\right)\in R^{C\times N\times D}$$
  where *D* is the embedding dimension.
- **Spectral Stream:** Recognizing that specific frequency bands (e.g., Gamma, Alpha) are biomarkers for emotion, we incorporate an explicit Fourier transform branch. Since EEG spectral power typically follows a 1/f distribution, high-frequency features often have vanishingly small magnitudes compared to low-frequency bands. To address this domain discrepancy, we employ a Log-Magnitude scaling strategy:
  (2) $$E_{freq}=W_{freq}\cdot \log(1+|\mathrm{rFFT}\left(X_{in}\right)\left|\right)\in R^{C\times N\times D}$$
  Here, the log(1 + x) operation compresses the dynamic range, preventing low-frequency dominance while preserving high-frequency details. $W_{freq}$ is a learnable linear projection that maps the spectral features to the latent dimension *D*.

#### 3.1.2. Feature Fusion

The final embedding $H^{\left(0\right)}$ is the element-wise sum of both streams, augmented by a learnable positional encoding $P_{pos}$ to preserve the spatial topology of the electrode grid. Instead of manually assigning scalar weights, we rely on the learnable parameters in $\phi_{time}$ and $W_{freq}$ to automatically adjust the contribution of each domain during the training process:

(3) $$H^{\left(0\right)}=E_{time}+E_{freq}+P_{pos}$$

### 3.2. Spatio-Temporal Hybrid Mamba-Attention Architecture

The core challenge in applying 1D State Space Models to EEG is the *serialization ambiguity* of the 2D spatio-temporal tensor. Naive flattening disrupts either spatial correlations or temporal continuity. To resolve this, we propose a *Decoupled Spatial–Temporal Scanning* strategy, utilizing the efficient Mamba2 block as the backbone.

#### 3.2.1. Structured State Space Duality

We adopt the Mamba2 block [14], which unifies State Space Models (SSMs) and linear attention via the Structured State Space Duality (SSD). For a sequence input $u\in R^{L\times D}$, the block learns a mapping $y=\Phi_{\mathrm{SSM}}\left(u\right)$ via a hardware-efficient selective scan. We omit the specific derivation of SSD for brevity and focus on our topological adaptation.

#### 3.2.2. Decoupled Spatial–Temporal Scanning

Our key insight is to dynamically reconfigure the data tensor layout between layers to explicitly model orthogonal dependencies. We define two viewing perspectives for the latent tensor $H^{\left(l\right)}$:

**1. Spatial-First View:** In the initial stages, we prioritize capturing the functional connectivity between brain regions. We permute and flatten the tensor to prioritize the channel dimension *C* in the inner loop:

(4) $$V_{spatial}={\mathrm{Unfold}}_{C\to inner}\left(H^{\left(l\right)}\right)\in R^{B\times (N\cdot C)\times D}$$

The resulting sequence consists of *N* segments, where each segment contains *C* channel tokens at a specific timestamp. The SSM scan effectively learns the global spatial distribution at time $t_{i}$ before transitioning to $t_{i+1}$.

**2. Temporal-First View:** In subsequent layers, we transpose the tensor to prioritize the time dimension *N*:

(5) $$V_{temporal}={\mathrm{Unfold}}_{N\to inner}\left(H^{\left(l+k\right)}\right)\in R^{B\times (C\cdot N)\times D}$$

Here, the sequence represents *C* independent time series. The SSM scan tracks the emotional evolution within a single channel over the entire duration before moving to the next channel, effectively isolating temporal dynamics from spatial interference.

Difference from Visual State Space Models: It is worth noting that while our scanning strategy shares structural similarities with recent Vision Mamba architectures (e.g., Vim, VMamba), the underlying motivation and implementation differ fundamentally due to the domain-specific physics of EEG signals.

Visual models typically process images where the height and width dimensions are isotropic (both representing spatial distance). Consequently, they employ multi-directional scanning (e.g., four-way cross-scanning) primarily to ensure global receptive fields across a symmetric 2D plane.

In contrast, EEG data constitute an anisotropic spatio-temporal tensor. The channel dimension represents functional brain connectivity, while the time dimension represents causal neural dynamics. Our Decoupled Spatial–Temporal Scanning is not merely a geometric traversal but a physical disentanglement strategy. By strictly alternating between “Spatial-First” and “Temporal-First” views, we force the model to explicitly learn orthogonal representations: instantaneous network topology and continuous state evolution. Unlike the simultaneous 2D integration in vision models, our approach respects the distinct physical nature of the spatial and temporal axes in physiological signals.

#### 3.2.3. Bidirectional Flipping Mechanism

Since standard SSMs are causal, we employ a bidirectional strategy to capture non-causal dependencies essential for classification. For a given view V, the output is computed as:

(6) $$H_{out}=\Phi_{\mathrm{SSM}}\left(V\right)+\mathrm{Flip}\left(\Phi_{\mathrm{SSM}}\left(\mathrm{Flip}\left(V\right)\right)\right)$$

This allows the model to utilize context from both the past and the future.

### 3.3. Global Context Recalibration

While Mamba efficiently compresses history into a recurrent state, it may experience information decay over extremely long sequences. To ensure robust classification, we introduce a **Global Attention Hybrid** layer at the deep stage. We employ a standard Multi-Head Self-Attention (MHSA) module to perform a global comparison of all channel-time tokens:

(7) $$H_{final}=\mathrm{MHSA}\left(\mathrm{LN}\left(H^{\left(L\right)}\right)\right)+H^{\left(L\right)}$$

This layer acts as a global recalibration mechanism, allowing the model to directly attend to salient emotional events regardless of their temporal distance, compensating for the potential “forgetting” of the recurrent SSM.

### 3.4. Classification Head

Finally, the output feature map $H_{final}\in R^{B\times C\times N\times P}$ is flattened to $R^{B\times (C\cdot N\cdot P)}$. We employ a robust Multi-Layer Perceptron (MLP) classifier:

(8) $$y_{pred}=\mathrm{MLP}\left(\mathrm{Flatten}\left(H_{final}\right)\right)$$

The MLP consists of three linear layers with ELU activation and Dropout, mapping the high-dimensional feature vector to the class logits. We minimize the Label Smoothing Cross-Entropy Loss to mitigate overfitting on noisy EEG labels:

(9) $$L=-\sum_{i=1}^{K}\left(\left(1-\epsilon \right)y_{i}+\frac{\epsilon }{K}\right)\log\left(p_{i}\right)$$

where $y_{i}$ is the ground truth, $p_{i}$ is the predicted probability, and ϵ is the smoothing factor.

## 4. Experiments and Results

### 4.1. Dataset

The FACED dataset is a comprehensive compilation of 32-channel EEG data collected from 123 subjects watching 28 different emotion-inducing video clips. This dataset encompasses a wide range of emotions, including nine categories: amusement, inspiration, joy, tenderness, anger, fear, disgust, sadness, and a neutral state.

The SEED-V dataset is derived from 62-channel EEG recordings of 16 subjects. It prompts participants to experience five emotional categories: disgust, fear, sad, neutral, and happy.

### 4.2. Implementation Details

The signals were downsampled to 200 Hz to reduce computational redundancy while preserving spectral fidelity up to the Gamma band. Subsequently, the band-pass filter (0.3–75 Hz) was applied exclusively to the SEED-V dataset. The 0.3 Hz high-pass cutoff effectively removes baseline drift and low-frequency artifacts, while the 75 Hz low-pass cutoff mitigates high-frequency muscle activity (EMG) and line noise. We adopted an evaluation protocol, strictly separating the training, validation, and testing sets to ensure the model generalizes. For the FACED dataset, trials from the first 80 subjects were used for training, the next 20 for validation, and the remaining 23 for testing. For the SEED-V dataset, the first five trials were used for training, the next five for validation, and the remaining five for testing. The training program spanned 200 epochs, using the PyTorch 2.6 framework on a Geforce RTX 3090 GPU. The optimization algorithm of choice was AdamW, configured with a learning rate of 0.0001, a weight decay of 0.0001, and a batch size of 32.

### 4.3. Comparison Experiment

The results of the comparison between the STHMA and previous methods on the FACED dataset and SEED-V datasets are shown in Table 1 and Table 2. In summary, the STHMA demonstrated remarkable recognition accuracy of 51.36% and 37.61% on the FACED datasets (9 classes, chance level: 11.11%) and SEED-V datasets (5 classes, chance level: 20.00%), respectively. This improvement is attributed to the backbone’s ability to model continuous emotional dynamics without the tokenization artifacts inherent in Transformers.

### 4.4. Ablation Experiment

As shown in Table 3 and Table 4, the accuracy increases by 3.36% for the FACED dataset and by 6.14% for the SEED-V dataset using Dual-Domain Physics-Aware Embedding. The accuracy increases by 1.72% for the FACED dataset and by 0.21% for the SEED-V dataset using temporal domain embedding. The accuracy increases by 2.47% for the FACED dataset and by 0.2% for the SEED-V dataset using spectral domain embedding. The results confirm that the “Log-Magnitude” scaled spectral features and the normalized temporal features are complementary. Meanwhile, the accuracy increases by 4.49% for the FACED dataset and by 15.29% for the SEED-V dataset using the STHMA Backbone. This confirms our hypothesis that explicitly modeling instantaneous spatial connectivity before tracking temporal evolution is crucial for reconstructing the spatio-temporal manifold. In all components of the STHMA, the STHMA Backbone plays a more important role than Dual-Domain Physics-Aware Embedding.

### 4.5. Comparative Analysis of Computational Costs and Performance

To rigorously evaluate the trade-off between the computational efficiency of the Mamba2 backbone and the theoretical complexity introduced by the Global Multi-Head Attention layer, we conducted a comparative study between a pure Mamba2 model and our hybrid STHMA architecture. The results are summarized in Table 5.

While the inclusion of the attention mechanism theoretically introduces a quadratic complexity term ($O(N^{2})$), our empirical results demonstrate that the practical impact on computational cost is marginal. As shown in Table 5, compared to the pure Mamba2 baseline, the STHMA requires only a slight increase in parameters and inference latency.

However, this minor computational overhead yields a significant performance gain. The hybrid architecture improves Balanced Accuracy by 4.62% on FACED and 4.05% on SEED-V compared to the pure Mamba2 model. This indicates that the hybrid design effectively leverages Mamba2 for efficient feature extraction and Attention for global context recalibration. The resulting architecture maintains high inference speeds suitable for real-time BCI systems while achieving superior recognition accuracy, validating that the performance benefits of the hybrid structure far outweigh its additional computational costs.

### 4.6. Visualization and Interpretability

**Figure 2:**
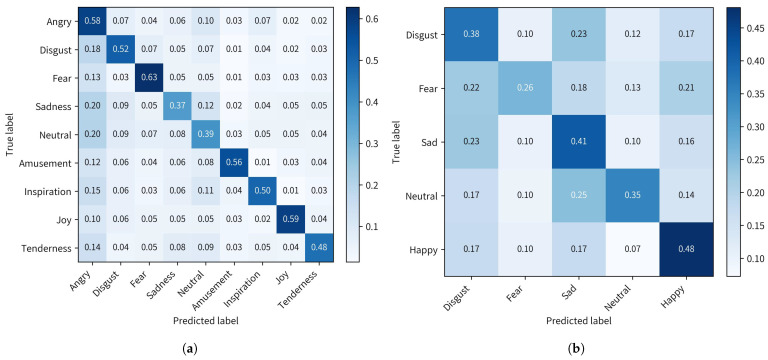
(**a**) The confusion matrix of the STHMA on FACED dataset. (**b**) The confusion matrix of the STHMA on SEED-V dataset.

To gain a granular insight into the recognition performance of the model, a confusion matrix was used to be an analytical tool. This matrix provides a detailed view of the results by illustrating the probability of accurate emotion classification along with the misclassification probabilities for each emotion relative to others. Figure 2 shows confusion matrices of emotion recognition generated by the STHMA on the FACED and SEED-V datasets.

## 5. Discussion

The empirical results demonstrate that the STHMA achieves state-of-the-art performance in decoding affective states from EEG. Beyond the numerical improvements, the success of our architecture offers significant theoretical insights into the modeling of physiological time series. In this section, we interpret these findings through the lenses of continuous dynamics, topological disentanglement, and inductive biases.

### 5.1. Modeling Continuous Neural Dynamics

In Transformer architectures, patches are treated as independent tokens in a set. The Self-Attention mechanism is inherently permutation-invariant, relying entirely on static positional encodings to infer order. This effectively treats the EEG signal as a discrete sequence of “snapshots” without an intrinsic mechanism to model the continuous flow of the underlying neural state.

Conversely, in the STHMA, the patching process serves a fundamentally different role: it acts as the discretization step size (Δ) for the underlying Ordinary Differential Equation (ODE), $h_{t}=\bar{A}h_{t-1}+\bar{B}x_{t}$. Unlike Transformers, where patches interact via global correlation, the STHMA processes patches via state recurrence. The hidden state $h_{t}$ carries the accumulated history from patch t−1 to patch *t*, strictly adhering to the causal evolution of the system. Therefore, even though the input is segmented for computational feasibility, the latent state trajectory remains continuous and physically grounded, preserving the dynamical properties of the neural signals.

### 5.2. Geometric Interpretation of the Decoupling Strategy

A central challenge in deep learning for BCI is the “Curse of Dimensionality” when processing 2D spatio-temporal tensors (Channels × Time). Standard 1D sequence models force a serialization that prioritizes one dimension at the expense of the other. Our *Decoupled Spatial–Temporal Scanning* strategy can be interpreted geometrically as learning via *Alternating Orthogonal Projections*.

- **The Spatial-First View** approximates the instantaneous *Functional Connectivity Networks*. By grouping channels at a frozen timestamp, the SSM captures the graph topology of brain regions co-activated during specific emotional stimuli.
- **The Temporal-First View** models the *Independent Component Dynamics*. By pivoting to scan along the time axis, the model isolates the temporal evolution of individual brain regions.

By alternating between these views, the STHMA effectively reconstructs the complex 3D spatio-temporal manifold using efficient 1D operators. This “Divide-and-Conquer” approach avoids the quadratic cost of full spatio-temporal attention while preserving the structural integrity of the EEG signal.

### 5.3. The Role of Physics-Aware Inductive Biases

Deep learning models often struggle with the non-stationarity of EEG signals—statistical properties that shift unpredictably over time. Our ablation studies confirm that the Dual-Domain Physics-Aware Embedding is not merely a feature augmentation, but a critical *Inductive Bias*. Raw time-domain convolution is adept at capturing transient Event-Related Potentials (ERPs), but it struggles to learn phase-invariant spectral features from limited data. By explicitly injecting Log-Magnitude spectral features, we anchor the model’s latent space with domain knowledge (i.e., the stability of Alpha/Beta bands in emotion). This dual-domain fusion ensures that the model is robust against temporal shifts, as spectral power is generally more stationary than raw waveform phase.

### 5.4. Synergy Between Mamba and Global Attention

An intriguing finding is the effectiveness of the hybrid architecture. While an SSM excels at compressing history into a fixed-size state, it theoretically suffers from a “bottleneck” when comparing information across very long distances. The inclusion of the Global Attention layer acts as a *Contextual Recalibration* mechanism. It allows the model to “look back” and directly compare salient emotional events (e.g., a burst of fear at t=1 s vs. t=4 s) without strictly relying on the recurrent state. This hybrid design balances the efficiency of selective scanning with the global reasoning capability of attention.

### 5.5. Limitations and Future Work

Despite these advancements, several limitations persist. First, while Mamba2’s parameters are efficient, the latent state dynamics are less interpretable than the explicit attention maps of Transformers. Developing visualization tools to map the SSM’s “state activation” back to scalp topography is a priority for clinical acceptance. Second, although our Cross-Subject results are promising, the performance gap between subject-dependent and subject-independent settings remains significant. Future work will explore large-scale *Self-Supervised Pre-training* of the STHMA on massive unlabeled EEG corpora to learn universal brain dynamic representations, potentially mitigating the calibration burden for new users.

## 6. Conclusions

In this work, we addressed the fundamental challenge of decoding affective states from non-stationary, high-dimensional EEG signals. We argued that traditional deep learning paradigms either struggle with long-range dependencies (CNNs/RNNs) or disrupt the continuous dynamical nature of physiological signals while incurring prohibitive computational costs (Transformers). To bridge this gap, we proposed the **Spatio-Temporal Hybrid Mamba-Attention (STHMA)**, a novel architecture that establishes Structured State Space Models as a viable and superior backbone for neurophysiological analysis.

Our methodological contributions center on adhering to the physical principles of neural data. By introducing a **Dual-Domain Physics-Aware Embedding**, we effectively mitigated the non-stationarity of EEG by anchoring representations in robust spectral features. Crucially, our **Decoupled Spatial–Temporal Scanning** strategy resolved the “serialization ambiguity” inherent in 1D sequence models. We demonstrated that explicitly disentangling the modeling of instantaneous brain connectivity from temporal emotional evolution is essential for reconstructing the complex spatio-temporal manifold of EEG.

The STHMA not only achieved state-of-the-art accuracy but also demonstrated superior computational efficiency, scaling linearly with sequence length. This confirms that coupling efficient continuous-state modeling with global attention refinement offers a robust path for handling the inter-subject variability of brain signals.

The STHMA opens new avenues for large-scale neurological computing. Its linear complexity eliminates the bottleneck for processing extremely long recordings, making it an ideal candidate for Self-Supervised Pre-training on massive unlabeled EEG corpora. We envision that scaling the STHMA towards a “Brain Foundation Model” will be a pivotal step in unlocking universal representations for diverse Brain–Computer Interface applications.

## Figures and Tables

**Table 1:** Comparison of STHMA and previous methods on FACED dataset (9 classes).

Methods	Balanced Accuracy (%)	Cohen’s Kappa (%)	Weighted F1 (%)
EEGNet	40.90 ± 1.22	33.42 ± 2.51	41.24 ± 1.41
EEGConformer	45.59 ± 1.25	38.58 ± 1.86	45.14 ± 1.07
SPaRCNet	46.73 ± 1.55	39.78 ± 2.89	47.29 ± 1.33
ContraWR	48.87 ± 0.78	42.31 ± 1.51	48.84 ± 0.74
CNN-Transformer	46.97 ± 1.32	40.17 ± 1.68	47.20 ± 1.25
FFCL	46.73 ± 1.58	39.87 ± 3.83	46.99 ± 1.45
ST-Transformer	48.10 ± 0.79	41.37 ± 1.33	47.95 ± 0.96
Icaps-ReLSTM	43.74 ± 0.41	36.48 ± 0.45	43.69 ± 0.42
MACTN	43.34 ± 2.83	36.00 ± 3.27	43.87 ± 2.90
Ours (STHMA)	51.36 ± 0.87	44.76 ± 1.12	51.52 ± 1.08

**Table 2:** Comparison of STHMA and previous methods on SEED-V dataset (5 classes).

Methods	Balanced Accuracy (%)	Cohen’s Kappa (%)	Weighted F1 (%)
EEGNet	29.61 ± 1.02	10.06 ± 1.43	27.49 ± 0.98
EEGConformer	35.37 ± 1.12	17.72 ±1.74	34.87 ± 1.36
SPaRCNet	29.49 ± 0.78	11.21 ± 1.39	29.79 ± 0.83
ContraWR	35.46 ± 1.05	19.05 ± 1.88	35.44± 1.21
CNN-Transformer	36.78 ± 0.78	20.72 ± 1.83	36.42± 0.88
FFCL	36.41 ±0.92	20.78 ± 2.01	36.45± 1.32
ST-Transformer	30.52 ±0.72	10.83 ± 1.21	28.33 ± 1.05
Icaps-ReLSTM	35.61 ± 0.56	19.46 ± 0.78	36.10 ± 0.69
MACTN	37.28 ± 0.90	21.61 ± 1.23	37.68 ± 1.10
Ours (STHMA)	37.61 ± 0.81	21.71 ± 1.56	37.40 ± 0.85

**Table 3:** The ablation experiments of the STHMA on the FACED dataset.

	Balanced Accuracy (%)	Cohen’s Kappa (%)	Weighted F1 (%)
w/o temporal domain embedding	49.64	42.69	48.33
w/o spectral domain embedding	48.89	41.90	48.10
w/o Dual-Domain Physics-Aware Embedding	48.00	40.90	47.80
w/o STHMA Backbone	46.87	40.02	47.15
Ours (STHMA)	51.36	44.76	51.52

**Table 4:** The ablation experiments of the STHMA on the SEED-V dataset.

	Balanced Accuracy (%)	Cohen’s Kappa (%)	Weighted F1 (%)
w/o temporal domain embedding	37.40	21.51	37.32
w/o spectral domain embedding	37.41	21.64	37.64
w/o Dual-Domain Physics-Aware Embedding	31.47	14.23	31.88
w/o STHMA Backbone	22.32	3.14	22.79
Ours (STHMA)	37.61	21.71	37.40

**Table 5:** Comparison of hybrid structure and pure Mamba2 structure on FACED dataset and SEED-V dataset.

Datasets	Methods	Balanced Accuracy (%)	Cohen’s Kappa (%)	Weighted F1 (%)	Params (M)	Runtime (ms)
FACED	pure Mamba2	46.74	40.12	47.86	1.26	8.64
	STHMA	51.36	44.76	51.52	1.42	10.63
SEED-V	pure Mamba2	33.56	16.73	33.52	1.26	6.79
	STHMA	37.61	21.71	37.40	1.42	7.92

## Data Availability

The data presented in this study are publicly available and can be accessed by submitting a request through the SEED-V dataset’s official website and the FACED dataset’s official website. For the SEED-V dataset, researchers are required to complete a license agreement to obtain access [https://bcmi.sjtu.edu.cn/home/seed/seed-v.html, accessed on 14 November 2025]. For the FACED dataset, please refer to the FACED download page [https://doi.org/10.7303/syn50614194].
